# Normal response inhibition in boys with Tourette syndrome

**DOI:** 10.1186/1744-9081-4-29

**Published:** 2008-07-18

**Authors:** Veit Roessner, Björn Albrecht, Peter Dechent, Jürgen Baudewig, Aribert Rothenberger

**Affiliations:** 1 Department of Child and Adolescent Psychiatry, University of Goettingen, Germany; 2 MR-Research, University of Goettingen, Germany

## Abstract

**Background:**

Inhibitory deficits are often a matter of debate in the pathophysiology of Tourette syndrome (TS). Previous neuropsychological studies on behavioral inhibition revealed equivocal results.

**Methods:**

To overcome existing shortcomings (e.g. confounders like medication status, comorbid conditions) we compared medication naïve boys (10–14 years) suffering exclusively from TS with age, gender and IQ matched healthy controls using a highly demanding Go/Nogo task that controls for novelty effects.

**Results:**

The performance did not differ between boys with TS and healthy boys.

**Conclusion:**

In TS normal response inhibition performance as measured by a Go/Nogo task can be assumed. However, there might be neurophysiological abnormalities in TS possibly related to compensatory mechanisms to control for tics. Hence, further studies combining neuropsychological and neurophysiological methods (e.g. electroencephalography, fMRI) using the same strictly controlled design along the whole range of development and tic severity are recommended.

## Background

Tourette syndrome (TS) is a neurodevelopmental disorder of childhood onset characterized by chronic motor and vocal tics. Although its etiology and pathophysiology are still unknown, there is increasing evidence for disruptions in the structure and function of cortico-striatal-thalamic-cortico (CSTC) neural circuitry. This altered functioning of CSTC circuitry including the prefrontal cortex might be associated with general problems of inhibitory control, not only of motor function, but also of cognitive and emotional regulation [1]. Unfortunately, the large number of neuropsychological studies addressing inhibition in TS could not draw a clear picture (for a review see [1,2]).

First, it has to be taken into consideration that there are separable types of inhibition, probably all with different neural substrates [3]. However, there were inconsistencies between studies in TS even when they used the same task for measuring inhibitory control. For example, a flanker task did not show deficits in response inhibition in adults [4], but in children with 'pure' TS compared to a control group [5]. Two studies using another test of response inhibition, an A-X version of the continuous performance task (CPT) revealed divergent findings in children with TS compared to healthy controls. One study found differences [6], the other not [7].

Second, all these inconsistencies might be attributable to several confounders. The uncontrolled psychiatric symptomatology often comorbid to TS like attention-deficit/hyperactivity disorder (ADHD) is thought to contribute mainly to found inhibitory deficits [8]. Additionally, previous and actual medication as well as short- and long-term compensatory mechanisms due to voluntary tic-suppression might have confounded previous findings on response inhibition performance in TS.

Hence, there is consensus that response inhibition in TS merits a more thorough investigation of less heterogeneous samples to come to firmer conclusions [4]. Because the a priori exclusion of confounders is the less biased method compared to post hoc statistical adjustment [9], we chose this approach to have the best ability to detect response inhibition deficits specific for TS per se. We applied a Go/Nogo task as one of the most common neuropsychological paradigms to investigate response inhibition in the motor domain [10,11].

Normal performance in the Go/Nogo task has been found in children with TS without comorbid conditions [12] as well as in adults with TS (some of them with comorbid ADHD and/or OCD) [13]. This is surprising as there are quite prominent differences between both versions of the Go/Nogo task applied.

For example in the task of Ozonoff et al. [12] the ratio of Go to Nogo stimuli is 50:50, whereas it is 83:17 in that of Hershey et al. [13]. Frequent Go stimuli are needed to ensure a prepotent tendency to respond that must then be inhibited for Nogo stimuli. But relatively rare Nogo stimuli entail the problem that novelty effects of increased arousal and orientation could not be distinguished from inhibitory requirements [14]. Hence, on the one hand a task with rare Nogo stimuli is needed to ensure that responses are prepotent and response inhibition is difficult, but on the other hand it has to control for the effects of stimulus probability [15]. This is even more important in TS as patients were found to be impaired on tasks requiring the processing of novel stimuli [16,17].

Hence, we progress the field of inhibitory control in TS in two points. First, we applied a more demanding Go/Nogo task than Ozonoff et al. [12] to detect inhibitory deficits that could possibly not be detected by their task. Additionally, the present Go/Nogo task controlled for the first time in TS research for the important confounding of novelty. Second, we included only medication naïve boys with 'pure' TS to avoid confounding effects of medication and psychopathology other than TS. To minimize the extent of compensatory mechanisms we included boys with a short history of tics. In accordance with previous studies on inhibitory control we expected impaired performance of the subjects suffering from TS compared to healthy controls in our highly demanding Go/Nogo task.

## Methods

### Subjects

Twenty-two medication naïve boys with TS, according to DSM-IV, were sequentially recruited from the outpatient clinic of the Department of Child and Adolescent Psychiatry of the University of Goettingen as well as via the homepage of the German Tourette Syndrome Association (see Table 1). Broadband psychopathology was screened by parent- and self-rated Strength and Difficulties Questionnaires (SDQ) as an internationally well established screening instrument of high psychometric quality [18]. Children with TS were rated on the Tourette Syndrome Severity Scale by a board certified child and adolescent psychiatrist (TSSS; [19]). Seventeen healthy boys were recruited as controls from a youth club in Goettingen. Subjects were aged 11 to 15 years, had normal or corrected to normal vision and understood task instructions as verified during practice trials. Full-scale IQ was estimated from the similarities, vocabulary, block design, and object assembly subtests of the Wechsler Intelligence Scale for Children (WISC-III; [20,21]). All children (cases and controls) were clinically assessed and best-estimate diagnoses were assigned on the basis of clinical observation, a semi-structured interview with parents and children (BADO; [22]) and various clinical ratings (parents, teachers, experts) (e.g. Conners Rating Scale; [23]), The Leyton Obsessional Inventory (LOI; [24])). All boys except for one boy from the TS group were right handed. All diagnoses were verified in a case conference by senior board-certified child psychiatrists. These experts have been working in clinical and research settings for TS and ADHD/obsessive-compulsive disorders (OCD) for many years. Informed consent was obtained from all subjects and their parents participating. The study had medical-ethical approval by the local ethics committee and was in accordance with the Helsinki Declaration.

**Table 1:** Sample description (Boys only)

**Measure**	**Healthy** **Comparison** ** N = 15**	**Tourette ** **Syndrome** ** N = 20**	**ANOVA**
	Mean (SD)	Mean (SD)	**F**_(1,33)_
Age (months)	153.3 (19.2)	150.0 (18.6)	0.3
Estimated Total IQ	107 (10.9)	108 (13.9)	0.1
TSSS ^a^	[0 (0)]	1.9 (1.6)	27.5**
Duration of tics (years) ^a^	[0 (0)]	5.7 (2.3)	128.3**
SDQ ^b^			
Self rated			
- *Total problems*	7.3 (4.0)	9.8 (5.5)	2.3
- *Emotional symptoms*	1.0 (1.3)	2.1 (1.5)	4.7*
- *Conduct Problems*	1.6 (1.1)	1.9 (1.4)	0.4
- *Hyperactivity*	3.3 (1.8)	3.7 (2.7)	0.3
- *Peer Problems*	1.4 (1.2)	2.2 (2.3)	1.3
- *Prosocial Behavior*	7.5 (1.5)	7.6 (1.9)	0.1
Parent rated			
- *Total problems*	4.9 (3.1)	10.0 (6.0)	8.8**
- *Emotional symptoms*	0.9 (1.0)	1.7 (2.0)	1.8
- *Conduct Problems*	0.7 (0.7)	2.4 (1.7)	12.3**
- *Hyperactivity*	3.1 (2.0)	4.3 (2.4)	2.1
- *Peer Problems*	0.3 (0.6)	1.7 (2.0)	7.0*
- *Prosocial Behavior*	8.3 (1.2)	7.1 (2.1)	3.7^+^

^a ^All children in the Healthy comparison group scored zero on the TSSS and Duration of tics. Thus, it was tested whether the score of the Tourette syndrome group differed from zero (df = 1, 19)

^b ^SDQ = Strength and Difficulties Questionnaire

^+ ^p < .1

* p < .05

** p < .01

In order to ensure validity of the task and to avoid confounding by insufficient compliance, two boys from each group had to be excluded because of insufficient performance (< 80% correct Go responses and/or < 33% correct rejections in the Nogo condition). The exclusion-rate did not differ between groups (χ^2 ^_(1) _= 0.1, p = .79).

### Behavioral Task and Experimental Procedures

The Go/Nogo task consisted of three runs with 150 trials each and was similar to that used by Tamm et al. [25]. White Go stimuli letters "X" (4/6 of all trials, i.e. frequent) and "A" (1/6, i.e. infrequent) as well as the Nogo stimuli letters "B" (1/6) were presented against a dark grey background in the centre of a 21" monitor at a viewing angle of approximately 2 degrees in a pseudo randomized order for 200 ms with an inter stimulus interval of 2000 ms. Subjects were instructed to press a button with the forefinger of their right hand to letters "X" and "A", but to withhold their response if a "B" occurred. Speed and accuracy were equally emphasized, and practice trials were administered as required.

### Data Analysis

Main dependent variables were (1) success rate (button press for Go stimuli "X", "A" and correct rejection of Nogo stimuli "B"), (2) response time for correct hits (RT of button press to Go stimuli) and (3) intra-individual variability of response time for correct hits (RT-SD). These were analyzed with repeated measures ANOVAs with within subjects factors "Run" (each of the three runs applied, which reflects time-on-task effects) and "Letter" (letters X, A, B, and thus Nogo or novelty effects) and between subjects factor "Group" (subjects suffering from TS vs. healthy controls). Since Go/Nogo performance is susceptible to developmental effects, all analyses were also conducted with age entered as a covariate. In order to correct for violations from sphericity, Greenhouse-Geisser ε and adjusted p-values are reported along with original degrees-of-freedom. Significant main effects were further explored using Sidak-adjusted post-hoc test. In case of interactions additional ANOVAS separately for each level of the respective factors were conducted. Significance level was set to 5%.

## Results

Success rate was different for the letters presented ("Letter" F_(2,66) _= 247.3, ε = .51, p < .01), with lower success rates for Nogo compared to both Go stimuli (see Table 2). Neither time on task effects as reflected by the factor "Run" or group-differences, nor any interactions were found.

**Table 2:** Go/Nogo-Task Performance (Boys only)

**Measure**	**Healthy ** **Comparison** ** N = 15**	**Tourette** **Syndrome** ** N = 20**	**repeated measure ANOVA** **(Sidak-adjusted Post-hoc tests)**^a^
	Mean (SD)	Mean (SD)	
*Success rate (%)*^b^			Letter: F_(2,66) _= 247.3**, (X > B**, A > B**)
*Go (X)*^c^	98 (1.8)	97 (2.1)	
*Go (A)*^d^	98 (2.1)	98 (2.4)	
*Nogo (B)*^e^	61 (17.1)	60 (11.7)	
*Reaction time of correct * *responses (ms)*^*b*^			Letter: F_(1,33) _= 66.8**, (A > X**)
*Go (X)*	401 (106)	378 (44)	
*Go (A)*	438 (106)	403 (59)	
*Reaction-time of errors compared to correct* * Go (A) (ms)*^f^			Letter: F_(1,33) _= 10.8**, (A > B**)
*Correct Go (A)*	434 (106)	404 (59)	
*Error in Nogo (B)*	372 (203)	347 (87)	
*Reaction-time variability (ms)*			Letter: F_(1,33) _= 13.2**, (A > B**)
*Go (X)*	109 (34)	118 (35)	Run*Group: F_(2,66) _= 2.8^+^
*Go (A)*	137 (39)	133 (49)	Run*Letter: F_(2,66) _= 3.2*

^a ^only effects with p < .10 are reported

^b ^marginal means across runs and standard deviations of factor "Group"

^c ^Go (X) was frequent (4/6 of all trials)

^d ^Go (A) was infrequent (1/6 of all trials)

^e ^Nogo (B) was infrequent (1/6 of all trials)

^f ^reaction-times of all three runs collapsed into a grand mean, thus slight differences to the marginal means reported for analysis of 'Reaction time of correct responses (ms)' emerge.

^+ ^p < .1

** p < .01

RTs of correct responses were slower for "A" compared to "X" ("Letter" F_(1,33) _= 66.8, p < .01). Again, no effects of time on task or "Group" or any interactions reached significance.

Reaction-times of Nogo errors were faster than correct responses to novel Go stimuli (F_(1,33) _= 10.8, p < .01), but did not differ between groups (F_(1,33) _= 0.6, p = .42) nor show an interaction "Letter*Group" (F_(1,33) _= 0.1, p = .90).

Intra-individual RT-SD of correct responses was lower on letters "X" compared to "A" ("Letter" F_(1,33) _= 13.2, p < .01), but there were also an interaction "Run*Letter" (F_(2,66) _= 3.2, ε = .99, p = .05) and a trend for an interaction "Run*Group" (F_(1,33) _= 2.8, ε = .96, p = .07). The latter trend was disentangled by considering confidence intervals at p < .10. In the first run controls displayed higher RT-SD than boys with TS which reversed in the third block where subjects suffering from TS showed higher RT-SD; in the second run no group differences were found. No general main effects of "Group" were found (F_(1,33) _= 0.1, p = .86). Separate repeated measure ANOVAs for each Go stimulus revealed no effects for letter "A", but an interaction "Run*Group" for letter "X" (F_(2,66) _= 4.5, ε = .99, p = .02) that was accompanied by a trend for higher RT-SD for subjects suffering from TS in the last run only. All these results remained stable also after entering age as a covariate to test for developmental effects.

## Discussion

Using a Go/Nogo task we found no differences in response inhibition between drug naïve boys suffering from TS without any comorbid disorder and healthy controls of same age, gender and IQ. The high demanding Go/Nogo task applied here following Tamm et al. [25] controlled for the first time in TS research for novelty effects in the Go/Nogo task. Additionally, the inclusion of only boys with 'pure' TS who had never taken psychotropic medication is a further methodological progress of previous work [12]. The higher scores in some of the SDQ scales of the boys with TS have no relevance since all are in the lower normal range. This indicates that there are no comorbid symptoms or diagnoses. This in turn raises the question if studying the minority of patients with 'pure' TS also allows conclusions concerning TS in general, because TS is frequently (up to 90%) co-existing with further psychiatric problems [26]. In terms of the most frequently comorbid condition in TS, i.e. ADHD several studies on different domains (psychopathology, neuropsychology etc.) have shown that TS and ADHD co-occur in an additive manner [8,27,28]. Hence, it seems likely that 'pure' TS and TS comorbid with other conditions are very similar if not identical.

The fact that RTs to the infrequent Go stimuli "A" were slower than to the frequent Go stimuli "X" highlights the importance of controlling for novelty effects. Furthermore, the task used in this study is highly demanding as reflected by the higher percentage of false alarms (~60%) compared to that of the tasks in the studies of Ozonoff (~15%; [12]) and Hershey et al. (~20%; [13]). Nevertheless, the present results based on a TS sample excluding possible confounders like gender, medication and comorbidity are in line with previous less controlled studies which reported no group differences in Go/Nogo performance of children [12] and adults with TS [13]. Thus they add evidence to the increasing base of knowledge that in 'pure' TS no deviances in neuropsychological task performance can be found except some few specific deficits. One of the latter might be a deficit of patients with TS in tasks requiring higher inhibition capacities [29]. Another one might be indicated in the present study by a trend for group differences in time on task effects for RT-SD. However, these exploratory findings of time on task effects require replication by more appropriate task designs to allow interpretation.

However, it has to be noted that the severity of TS was moderate in our group as indicated by the TSSS [19] and SDQ parent- and self-rated scores [18]. Hence, assuming a correlation between tic severity and inhibitory deficits one could speculate that patients with more severe tics might show performance deficits in inhibitory tasks. But this speculation is questioned by the findings of Mueller et al. [30] who showed that chronic suppression of tics results in an enhancement of the executive processes involved in inhibitory control. Hence, the question arises if more severe tics would result in a more efficiently trained inhibitory control than milder tics.

However, the absence of performance differences in a Stop-task between a group with TS and healthy controls seems to be in contrast to the differences in co-registered neurophysiological parameters between these groups during the same experiment. The process of response inhibition was related to an enhanced frontal brain electrical negativity and a more anterior scalp distribution of the Nogo-anteriorization component in TS [31]. Similarly, Serrien et al. [32] interpreted an elevated frontomesial electroencephalographic coherence in TS as serving to compensate for diminished inhibitory control in a special Go/Nogo task.

Hence, compensatory mechanisms in TS to control for tics might lead to neurophysiological deviances but yield normal findings in neuropsychological performance on response inhibition in TS. To better understand the relationship between neuronal mechanisms of response inhibition and its neuropsychological performance in TS further studies combining neuropsychological and neurophysiological methods (e.g. electroencephalography, fMRI) using the same strictly controlled design along the whole range of development and tic severity are recommended.

## Competing interests

The authors declare that they have no competing interests.

## Authors' contributions

VR designed and coordinated the study and drafted the manuscript. BA has been responsible for the data analysis and contributed to the interpretation of the results. PD, JB participated in the acquisition of data, discussions about the data analyses and commented on the written drafts of the manuscript. AR participated in the overall conceptualization and supervision of project, including the design and interpretation of the results. All authors have read and approved the final manuscript.
